# Are serum MOTS-c levels and MOTS-c m.1382A>C polymorphism related to polycystic ovary syndrome?

**DOI:** 10.20945/2359-4292-2026-0031

**Published:** 2026-04-01

**Authors:** Berna Eroğlu Filibeli, Fatima Dedemoglu, Pinar Garipçin, Seyran Bulut, Banu İşbilen Başok, Sefa Kizildağ, Bumin Dündar, Gönül Çatli

**Affiliations:** 1 Izmir City Hospital, Department of Pediatric Endocrinology, Izmir, Turkey; 2 Dokuz Eylul University, Department of Medical Biology and Genetics, Izmir, Turkey; 3 Erciyes University Faculty of Medicine, Department of Pediatric Rheumatology, Kayseri, Turkey; 4 Hitit University Corum Erol Olçok Training And Research Hospital, Department of Pediatric Endocrinology, Corum, Turkey; 5 University of Health Sciences, Dr. Behcet Uz Children’s Training & Research Hospital, Department of Medical Biochemistry, Izmir, Turkey; 6 Izmir Katip Celebi University, Department of Pediatric Endocrinology, Izmir, Turkey; 7 Istinye University, Faculty of Medicine, Department of Pediatric Endocrinology, Istanbul, Turkey

**Keywords:** MOTS-c, mitochondrial DNA, polymorphism, polycystic ovary syndrome, adolescent

## Abstract

**Objective:**

MOTS-c is a mitochondria-derived peptide associated with reduced insulin resistance and obesity. The m.1382A>C polymorphism of the *MOTS-c* gene is linked to an increased risk of type 2 diabetes in men. However, no studies have explored the relationship between this polymorphism and MOTS-c levels in adolescents with polycystic ovary syndrome (PCOS). This study aimed to investigate the differences in MOTS-c levels between adolescents diagnosed with PCOS and those without PCOS, as well as the associations with metabolic parameters. The association between the *MOTS-c* gene polymorphism and serum MOTS-c levels in adolescents with PCOS was also evaluated.

**Subjects and methods:**

Adolescents aged 12-18 diagnosed with PCOS were recruited based on irregular menstrual cycles and clinical/biochemical hyperandrogenism, excluding other conditions. The control group consisted of adolescents with regular menstruation. Serum MOTS-c levels were measured using ELISA, and the m.1382A>C polymorphism was analyzed by sequencing.

**Results:**

The study included 121 adolescents with PCOS and 125 healthy controls. The mean serum MOTS-c levels in the PCOS group were higher than in the control group; however, this difference did not reach statistical significance (p = 0.059). There was no significant association between MOTS-c levels and anthropometric or metabolic parameters within the PCOS group (p > 0.05). All participants had the wild-type (A/A) genotype for the m.1382A>C polymorphism.

**Conclusion:**

**Results:**

indicate that the *MOTS-c* gene (m.1382A>C) polymorphism shows no significant association with PCOS, and serum MOTS-c levels are comparable between individuals with PCOS and healthy controls, suggesting that MOTS-c may have a minor involvement in the pathophysiology of PCOS.

## INTRODUCTION

Polycystic ovary syndrome (PCOS) is a common endocrine and metabolic disorder that typically manifests during adolescence. PCOS is thought to affect from 5% to 10% of women of reproductive age worldwide (^1^). This is strongly associated with metabolic abnormalities such as insulin resistance, hyperinsulinemia, type 2 diabetes, hypertension, dyslipidemia, and infertility (^2^). Obesity in individuals with PCOS further exacerbates the risk of these metabolic comorbidities. Insulin resistance and hyperandrogenism are believed to be interrelated and may serve as central contributors to PCOS pathogenesis (^3^). Although the exact etiology of PCOS remains unclear, familial aggregation suggests a genetic component. The underlying etiology of PCOS is still being elucidated. An autosomal dominant pattern was initially proposed, based on the high prevalence of PCOS among first-degree relatives of affected individuals (^4^). However, twin studies emphasize the X-linked or polygenic inheritance (^5^,^6^). Thus, PCOS is now considered a complex and highly heritable disorder (^7^).

Mitochondria play a fundamental role in essential cellular processes, including metabolism, growth, and apoptosis, which are governed not only by nuclear DNA but also by signals encoded within mitochondrial DNA (mtDNA) (^8^). The interplay between nuclear and mitochondrial genomes, referred to as mitonuclear communication, has been increasingly recognized as a key regulatory mechanism. Among the molecules encoded by mtDNA are mitochondrial-derived peptides (MDPs), including humanin, MOTS-c (mitochondrial open reading frame of the 12S rRNA-c), and small humanin-like peptides (SHLPs). MOTS-c is a 16-amino acid peptide encoded within the 12S rRNA region, and it plays a critical role in maintaining insulin sensitivity and metabolic homeostasis, primarily via activation of the AMP-activated protein kinase (AMPK) pathway (^9^). MOTS-c is expressed in tissues such as muscle, brain, and liver and is also detectable in plasma (^10^). In mice studies, exogenous MOTS-c administration has been shown to improve insulin resistance, reduce adiposity, and mitigate obesity-related phenotypes (^9^,^11^). However, human studies investigating the metabolic effects of MOTS-c remain limited and show inconsistent findings (^10^,^12^,^13^). To date, only one study has explored the relationship between PCOS and MOTS-c, focusing on its response to intralipid and insulin infusion (^14^). Beyond circulating levels, genetic variation in the MOTS-c-encoding region (m.1382A>C; rs111033358) has been linked to altered metabolic risk in specific populations, potentially by modifying the peptide sequence and bioactivity (^15^,^16^). Given the metabolic disturbances characteristic of PCOS-including hyperinsulinemia, insulin resistance, obesity, and dyslipidemia-and the limited literature on MOTS-c, it was hypothesized that the m.1382A>C polymorphism and circulating MOTS-c levels may contribute to the pathogenesis of PCOS.

Therefore, this study aimed to compare the *MOTS-c* m.1382A>C polymorphism and serum MOTS-c levels in adolescents with and without PCOS and to evaluate the association of serum MOTS-c levels with metabolic and anthropometric parameters.

## SUBJECTS AND METHODS

A case-control study involving adolescent girls diagnosed with PCOS and a control group of adolescents with regular menstrual cycles was conducted. The local ethics committee approved the study (Date: 05.02.2020, Number: 2). Written informed consent was obtained from all participants and their legal guardians.

### Inclusion criteria

Adolescent girls aged 12 to 18 years diagnosed with PCOS, based on the existence of menstrual irregularity (oligomenorrhea) or anovulatory (secondary amenorrhea) cycles, and clinical or biochemical hyperandrogenism at least two years after menarche were enrolled in the PCOS group (^17^). The control group consisted of healthy volunteers with regular menstrual cycles for at least two years following menarche and without clinical signs of hyperandrogenism.

#### Diagnosis of PCOS in adolescents

Diagnosis of PCOS in adolescents was based on the following two criteria:

**A. Menstrual irregularity (oligomenorrhea) or anovulatory (secondary amenorrhea) cycles**
**B. Clinical or biochemical hyperandrogenism**
**Clinical hyperandrogenism**
    - Hirsutism was evaluated using the modified Ferriman-Gallwey score (FGS), with a score ≥ 8 considered as diagnostic for clinical hyperandrogenism (^1^).
**Biochemical hyperandrogenism**
    - Biochemical evaluation was performed in the 2^nd^ and 5^th^ days of the follicular phase between 08:00 h and 09:00 h. In cases without regular periods, a progesterone withdrawal test was used for timing of blood sampling (^18^).

Serum follicle-stimulating hormone (FSH), luteinizing hormone (LH), estradiol (E2), 17-hydroxy-progesterone (17-OH-PG), dehydroepiandrosterone sulfate (DHEA_SO4_), sex hormone binding globuline (SHBG), and total testosterone levels were measured in all cases with PCOS.

Due to the inability to measure free testosterone levels in the laboratory where the study was conducted, free androgen index [(total testosterone/SHBG) > 4.5] and total testosterone levels (> 55 ng/dL) were used as biochemical criteria for diagnosing hyperandrogenemia in patients with PCOS (^1^).

#### Pelvic ultrasonography (USG)

All participants underwent pelvic ultrasound between days two and five of the follicular phase, conducted by a single radiologist to exclude other pathologies that could mimic PCOS.

### Exclusion criteria

Exclusion criteria included girls under 12 or over 18 years; individuals less than two years post-menarche; those with chronic diseases (cardiovascular, gastrointestinal, respiratory, oncological, etc.); regular medication use, or endocrine conditions that could result in oligo/amenorrhea (e.g., thyroid disorders, pregnancy, primary ovarian failure, congenital adrenal hyperplasia, androgen-secreting tumors, Cushing’s syndrome, or hyperprolactinemia).

### Anthropometric assessment

Anthropometric measurements were conducted using a Harpenden stadiometer (Crosswell, Crymych, Pembs., SA41 3UF, UK), with a height measurement accuracy of 0.1 cm, and a SECA scale (Hammer Steindamm 3-25, 22089 Hamburg, Germany) with an accuracy of 0.1 kg for weight assessment. Participants were assessed wearing only light underwear to ensure accurate measurements. The Body Mass Index (BMI) was calculated by dividing weight (kg) by the squared height (m), and then converted into a standard deviation score (SDS) using national BMI reference data (^19^).

### Body fat analysis (bioimpedance analysis)

The bioelectrical impedance analysis method evaluated body fat mass (kg) and body fat percentage (Tanita BC-418, Tokyo, Japan).

### Blood pressure

Systolic (SBP) and diastolic blood pressures (DBP) were measured twice from the right arm using a calibrated sphygmomanometer after a 10-minute rest in the supine position. Blood pressure values higher than the 95^th^ centile for height, age, and gender were defined as hypertension (^20^).

### Metabolic syndrome (MS)

MS is defined by the International Diabetes Federation criteria for adolescents aged 10-16 years (^21^). It consists of abdominal or central obesity (90^th^ centile of waist circumference or adult cut-off if lower) plus at least two of the following features:

- Fasting plasma triglyceride (TG) ≥ 150 mg/dL;
- High-density lipoprotein cholesterol (HDL-C) < 40 mg/dL;
- Systolic blood pressure ≥ 130 mmHg and/or diastolic blood pressure ≥ 85 mmHg;
- Fasting plasma glucose ≥ 100 mg/dL, or known T2DM.

### Pubertal staging

Pubertal development was assessed by Tanner’s staging (^22^). Therefore, breast development Stage II or above is defined as puberty.

### Genetic analysis

Blood samples were taken from all subjects in an EDTA tube to study the *MOTS-c* gene variant. First, DNA was isolated using a commercial kit (DNA Isolation Kit for Mammalian Blood Roche) according to the manufacturer’s instructions. Genomic DNA quantity and purity were assessed by spectrophotometry (A260/A280 ≈ 1.8-2.0) and integrity was verified by agarose gel electrophoresis. Only samples meeting these thresholds were subjected to polymerase chain reaction (PCR) test. Then, the spectrophotometer and gel electrophoresis determined the purity and the quality of the isolated genomic DNA samples.

### Study protocol for determination of genotypes

SNP numbered *rs111033358* in the *MOTS-c* gene was evaluated. NCBI and Ensembl databases were used to access the gene sequence to examine the human *MOTS-c* gene. It was tested whether the forward and reverse primer sequences designed for the PCR reaction to be applied to this gene sequence were correctly seated. To identify the binding sites of the designed forward and reverse primer sequences on the genome and to assess their specificity, potential matches in the human genome were analyzed using the Primer-BLAST program capable of scanning the entire genome.

Conventional PCR testing was conducted with primers specific to the target gene region used in our study. Amplification success and specificity were confirmed by agarose gel electrophoresis, ensuring a single band of the expected amplicon size for each sample before sequencing. As a result, PCR products contain the SNP gene region numbered *rs111033358* in a large amount of the *MOTS-c* gene of each case.

DNAse and RNAse-free sterile distilled water were added to the lyophilized forward and reversed primers and the final concentration was adjusted to 100 pmol/µL. After the primer stocks were aliquoted, they were diluted at 1:10 with distilled water to be used in the PCR testing. While preparing the PCR reaction, all procedures were carried out on the cold block. A mixture containing *MOTS-c* gene-specific primer pairs was used for PCR reaction of genomic DNA whose purity and amount were determined. Then, the master mix was added to the isolated DNAs and placed in the thermal cycler. After the PCR program information was selected in the device, the obtained PCR products were checked by the gel electrophoresis method. Then, sequence analysis was performed with service procurement.

All PCR products were subjected to bidirectional Sanger sequencing at an accredited commercial facility (Macrogen, Seoul, South Korea). Raw electropherograms were independently inspected by two researchers using Chromas with manual verification of peak morphology and signal-to-noise at the polymorphic site (m.1382; rs111033358). Base calls were accepted only when forward and reverse reads were concordant and of adequate quality; discrepant or low-quality sites were reexamined and recalled after joint review.

**Figure 1:**
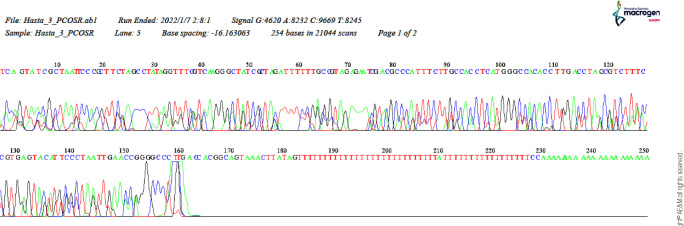
Sample image of DNA sequences in the Chromas program.

The sequences containing the *rs111033358* SNP gene region in the *MOTS-c* gene were read both ways by Macrogen^®^ (Seoul, South Korea) with forward and reverse primers. The data obtained were shared with our laboratory as DNA sequences by the company. The shared data were evaluated individually in the Chromas program as a graphic (**Figure 1**).

### Biochemical analysis

Venous blood samples were collected into plain blood collection tubes (BCT)s (BD Vacutainer^®^ SST II Advance Tube, 5 mL, 13 x 100 mm, USA). Serum samples were separated from cellular fragments by centrifugation for 10 minutes at 1.500 g within one hour after blood sampling. Serum samples were aliquoted and stored at -80 °C until further analysis.

Fasting serum glucose, total cholesterol (TC), TG, low-density lipoprotein-cholesterol (LDL-C), and HDL-C and alanine transaminase (ALT) levels were measured in Beckman AU5800 (Beckman Coulter, Brea, CA, USA). Serum free thyroxine (fT4), thyroid stimulating hormone (TSH), total testosterone, insulin, prolactin, FSH, LH, E2, and DHEA_SO4_ levels in DxI 800 immunoassay systems (Beckman Coulter, Brea, CA, USA) by using original in vitro diagnostic reagents. 17-OH-PG levels were analyzed using a RIA kit (17α-hydroxyprogesterone-RIA-CT; DIAsource ImmunoAssays S.A, Belgium) by following manufacturer’s instructions. The reference range for total testosterone was 8.4-48.1 ng/dL.

Serum MOTS-c concentrations were determined by a commercial kit employing the quantitative ELISA technique (Cloud-Clone Corp., TX, USA) (Catalog No: CEX132Hu, Lot No: L210719103). The analysis was carried out according to the manufacturer’s instructions. All ELISA measurements were run in duplicate; duplicate agreement met the pre-specified criterion (≤ 15% CV) for all samples. The kit’s intraand inter-assay coefficient variations (CVs) were < 10% and <12%, respectively. The test’s detection limit (LOD) value was less than 0.97 ng/mL. The measurement range of the assay was 2.47 to 200 ng per mL.

The cut-off points used to define dyslipidemia (> 95^th^ percentile values in healthy children for TC, TG, LDL-C, and < 5^th^ percentile value for HDL-C) were: TC > 200 mg/dL; TG > 150 mg/dL; LDL-C >130 mg/dL; HDL-C < 40 mg/dL (^23^).

The pathological criterion of impaired fasting glucose >100 mg/dL was used (^24^). Moreover, the “Homeostasis model assessment-insulin resistance” (HOMA-IR) index was used to evaluate insulin resistance. HOMA-IR was calculated as fasting insulin (µIU/mL) × fasting glucose (mg/dL)/405. A HOMA-IR value ≥ 4 was accepted as insulin resistance since all participants included in the study were pubertal (^25^). Pathological cut-off values for some biochemical parameters were as follows: TSH > 5 IU/L, ALT > 25 IU/L, 17-OH-PG > 2 ng/mL, total testosterone > 55 ng/dL (^26^), and prolactin > 25 ng/mL (^27^).

### Sample size

The G*Power 3.1 program was used to calculate the sample size with a power >80% at a 5% significance level. The calculation was based on the study by Ramanjaneya and cols. (^14^), which assessed plasma MOTS-c levels in adult women with PCOS. According to the results of the power analysis, a minimum of 204 participants (102 PCOS, 102 controls) were required to detect a 10% difference in the frequency of the *MOTS-c rs111033358* polymorphism between the PCOS and control groups and to observe a mean difference of 86.37 ng/mL in serum MOTS-c levels.

### Statistical analysis

All statistical analyses were conducted using SPSS version 24.0 (IBM Corp., Armonk, NY, USA). Before comparisons, data distribution was assessed using the Kolmogorov-Smirnov test, Q-Q plots, histograms, and evaluation of skewness and kurtosis. Descriptive statistics summarized demographic and clinical characteristics. Group proportions were compared with the Chi-squared test. For continuous variables, normally distributed data were analyzed with the Student’s *t*-test, whereas non-normally distributed data were evaluated using the Mann-Whitney *U* test. Differences in serum MOTS-c levels across subgroups (PCOS with obesity, PCOS without obesity, controls with obesity, and controls without obesity) were assessed using one-way analysis of variance (ANOVA). Owing to non-homogeneous distributions, correlations between serum MOTS-c and anthropometric or metabolic parameters were evaluated with Spearman’s correlation (ρ). Normally distributed variables are expressed as mean ± standard deviation (SD), and non-normally distributed variables as median [interquartile range (IQR)]. In addition to *p*-values, effect sizes were calculated to better convey the magnitude of group differences and associations: for *t*-tests, Cohen’s *d* was computed; for Mann-Whitney *U* tests, the effect size *r* was calculated as *Z*/√*N*. For correlation analyses, 95% confidence intervals (CIs) were estimated via bias-corrected and accelerated (BCa) bootstrapping with 1,000 resamples. Effect sizes were interpreted according to Cohen’s criteria (*r* < 0.10 negligible, 0.10-0.29 small, 0.30-0.49 medium, ≥ 0.50 large). A two-sided *p* < 0.05 was considered statistically significant.

## RESULTS

### Demographic parameters

A total of 121 adolescent girls with PCOS (median age: 16.2 years) and 125 healthy controls (median age: 15.7 years) were included in the study. The median birth weights were 3,300 g in the PCOS group and 3,350 g in the control group, with gestational ages of 40.0 and 39.0 weeks, respectively.

Among the PCOS participants, 68% (n = 85) had menstrual irregularities, 25.6% (n = 32) with hirsutism, and 6.4% (n = 8) for other complaints. Hirsutism was observed in 61.6% (n = 77) of the PCOS group, whereas no hirsutism was identified in the control group.

Obesity was observed in 41.6% (n = 52) of the PCOS group and 14.9% (n = 18) of the controls; small for gestational age (SGA) births were recorded in 12% (n = 12) of the PCOS and 6.6% (n = 8) of the control group. A positive family history of PCOS was reported in 20.8% (n = 26) of the PCOS group and 9.9% (n = 12) of the control group.

### Anthropometric, metabolic, and radiological parameters

Anthropometric data of adolescents with PCOS, BMI, BMI-SDS, waist circumference, fat mass, fat percentage, and SBP values were significantly higher than the control group (**Table 1**).

**Table 1:** Demographic and anthropometric characteristics and serum MOTS-c levels of the PCOS and control groups

	PCOS group (n = 121)	Control group (n = 125)	p	Effect size *r*
Age (years)	16.2 (1.6)	15.6 (2.3)	**0.026^a^**	**0.14**
BMI (kg/m^2^)	25.6 (8.2)	21.3 (6.1)	**<0.001^a^**	**0.37**
BMI-SDS	1.43 (2.02)	0.05 (2.38)	**<0.001^a^**	**0.35**
Waist circumference (cm)	79 (18.2)	67.0 (13.0)	**<0.001^a^**	**0.43**
Fat percentage (%)	32.9 ± 8.3	26.7 ± 8.5	**<0.001^b^**	**0.35**
Fat mass (kg)	23.3 (14.8)	13.9 (13.1)	**<0.001^a^**	**0.37**
SBP (mm/Hg)	110 (10.0)	110 (^5^)	**0.001^a^**	**0.23**
DBP (mm/Hg)	70 (10.0)	70 (10.0)	0.960^a^	< 0.1
Serum MOTS-c (ng/mL)	93.1 (146.6)	66.2 (115.3)	0.059^a^	0.12

a Mann-Whitney U test;

b Student’s t-test.

Data are presented as mean ± SD or median (IQR).

BMI: body mass index; SDS: standard deviation score; SBP: systolic blood pressure; DBP: diastolic blood pressure.

**Figure 2:**
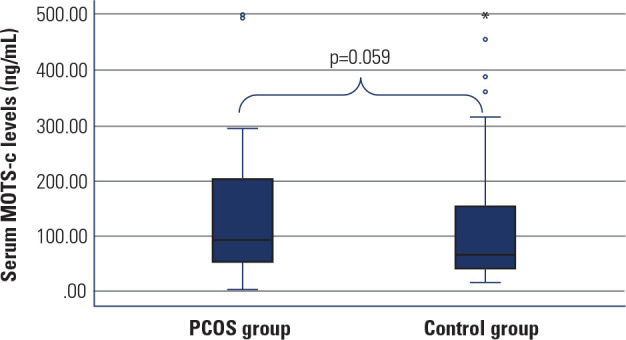
Serum MOTS-c levels of PCOS and control groups.

**Figure 3:**
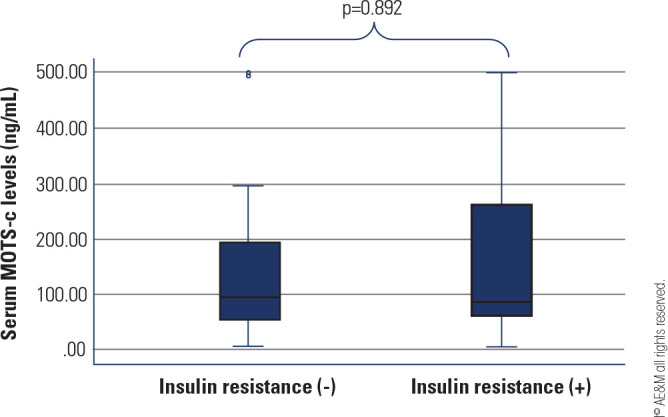
Serum MOTS-c levels according to insulin resistance in patients with PCOS.

The median serum MOTS-c level in the PCOS group was higher than in the control group (93.1 vs. 66.2 ng/mL), with a trend toward significance (p = 0.059) (**Table 1** and **Figure 2**). Among the PCOS group, there was no significant difference in the median serum MOTS-c levels of the adolescents with (n = 30) and without insulin resistance (n = 95) [84.1 (205.4) ng/mL & 93.0 (140.5) ng/mL, respectively; p = 0.892] (**Figure 3**). Participants were stratified into four subgroups: (^1^) adolescents with obesity in the PCOS group (n = 51), (^2^) adolescents without obesity in the PCOS group (n = 74), (^3^) adolescents with obesity in the control group (n = 19), and (^4^) adolescents without obesity in the control group (n = 102). One-way ANOVA revealed no significant difference in serum MOTS-c levels between these subgroups (p = 0.268).

The prevalence of impaired fasting glucose, insulin resistance, and subclinical hypothyroidism were 2.4%, 24%, and 4.8%, respectively, in the PCOS group. The frequencies of elevated TG, TC, LDL-C, ALT, and reduced HDL-C were 16.8%, 18.4%, 12%, 15.2%, and 16%, respectively. Total testosterone was elevated in 73.6% of adolescents with PCOS.

PCOS group was compared regarding metabolic parameters according to the presence of obesity: fasting insulin, HOMA-IR, ALT, TG, TC, and LDL-C levels were significantly higher in adolescents with obesity in the PCOS group. However, no significant difference in serum MOTS-c levels was observed between adolescents with and without obesity in the PCOS group (101.0 vs. 80.9 ng/mL, p = 0.160) (**Table 2**).

**Table 2:** Metabolic parameters of the PCOS group according to obesity

	PCOS group		p	Effect size *r*
	Adolescents with obesity (n = 52)	Adolescents without obesity (n = 73)		
Fasting glucose (mg/dL)	87.0 (9.0)	87.0 (9.0)	0.950^a^	0.01
Fasting insulin (mU/L)	17.6 (11.1)	10.4 (7.0)	**<0.001^a^**	**0.44**
HOMA-IR	3.9 (1.9)	2.3 (1.5)	**<0.001^a^**	**0.45**
ALT (IU/L)	17.0 (9.75)	14.0 (9.0)	**0.009^a^**	**0.23**
TG (mg/dL)	112.5 (72.0)	74.0 (28.5)	**<0.001^a^**	**0.45**
TC (mg/dL)	186.9 ± 32.5	157.5 ± 33.1	**<0.001^b^**	**0.41**
LDL-C (mg/dL)	113.6 ± 29.6	90.0 ± 25.0	**<0.001^b^**	**0.40**
HDL-C (mg/dL)	47.0 (11.0)	49 (^15^)	0.096^a^	0.15
Serum MOTS-c (ng/mL)	101.0 (187.5)	80.9 (151.0)	0.160^a^	0.13

a Mann-Whitney U test;

b Student’s *t*-test.

Data are presented as mean ± SD or median (IQR).

HOMA-IR: homeostasis model assessment-insulin resistance; ALT: alanine transaminase; TG: triglyceride; TC: total cholesterol; LDL-C: low-density lipoprotein-cholesterol; HDL-C: high-density lipoprotein-cholesterol.

No significant differences in LH, FSH, LH/FSH ratio, E2, total testosterone, progesterone, DHEA_SO4_, or 17-OH-PG were observed between adolescents with and without obesity in the PCOS groups, except for progesterone levels.

Metabolic syndrome was present in 11 (21.5%) adolescents with obesity in the PCOS group. Serum MOTS-c levels were comparable between adolescents with and without metabolic syndrome in the PCOS group [98.7 (439) vs. 101.8 (191.7) ng/mL; p = 0.889].

No significant correlation was observed between the PCOS group’s serum MOTS-c level and any of the anthropometric or metabolic parameters. Furthermore, after correcting for age, BMI, and BMI-SDS with partial correlation analysis, no correlation between serum MOTS-C level and anthropometric or metabolic parameters was found (**Table 3**).

**Table 3:** The relation of serum MOTS-c level with anthropometric and metabolic parameters in the PCOS group

	PCOS group (n = 125)		
	Spearman’s correlation (ρ)	^*^p	^*^95% CI (BCa)
Age (years)	0.001	0.987	[-0.20, 0.19]
BMI (kg/m^2^)	0.089	0.325	[-0.06, 0.29]
BMI-SDS	0.086	0.338	[-0.06, 0.29]
Waist circumference (cm)	0.057	0.537	[-0.11, 0.26]
Fat percentage (%)	0.058	0.542	[-0.12, 0.23]
Fat mass (kg)	0.074	0.435	[-0.10, 0.25]
Fasting glucose (mg/dL)	-0.175	0.051	[-0.34, 0.00]
Fasting insulin (mIU/L)	0.103	0.253	[-0.05, 0.29]
HOMA-IR	0.078	0.389	[-0.08, 0.27]
TG (mg/dL)	0.073	0.416	[-0.08, 0.27]
TC (mg/dL)	0.109	0.227	[-0.04, 0.30]
LDL-C (mg/dL)	0.105	0.246	[-0.05, .030]
HDL-C (mg/dL)	0.024	0.789	[-0.16, 0.21]
ALT (IU/L)	0.068	0.448	[-0.12, 0.22]
LH (mIU/L)	0.043	0.631	[-0.15, 0.22]
FSH (mIU/L)	-0.078	0.386	[-0.28, 0.09]
E2 (pg/mL)	-0.025	0.781	[-0.23, 0.16]
17-OH PG (ng/mL)	-0.039	0.667	[-0.27, 0.13]
Progesterone (ng/mL)	-0.138	0.125	[-0.38, 0.02]
Total Testosterone (ng/mL)	0.137	0.127	[-0.07, 0.33]

* Spearman correlations with BCa-bootstrapped 95% CIs (1,000 samples).

BMI: body mass index; SDS: standard deviation score; HOMA-IR: homeostasis model assessment-insulin resistance; TG: triglyceride; TC: total cholesterol; LDL-C: low-density lipoprotein-cholesterol; HDL-C: high-density lipoprotein-cholesterol; ALT: alanine transaminase; LH: luteinizing hormone; FSH: follicle stimulating hormone; E2: estradiol; 17-OH PG: 17-hydroxy progesterone.

### *MOTS-c* gene polymorphism

All participants in both groups had the wild-type genotype (A/A) for the m.1382A>C (rs111033358) SNP.

## DISCUSSION

Most women with PCOS are either overweight or obese. Studies report that about 80% of women with PCOS in the United States and 30%-50% in other countries are overweight or obese, 44%-70% have insulin resistance, and 21.4%-37% show impaired glucose tolerance (^28^,^29^). Dyslipidemia is characterized by elevated TC, TG, LDL-C, and, along with reduced HDL-C levels, it is highly prevalent, affecting up to 70% of women with PCOS (^30^). Moreover, biochemical hyperandrogenism is present in 60-80% of women with PCOS (^31^). In our adolescent cohort, we observed obesity in 42% of participants with PCOS, impaired fasting glucose in 2.4%, insulin resistance in 24% and biochemical hyperandrogenism in 76.6%. There were elevated TG, TC, and LDL-C levels in 16.8%, 18.4%, and 12% of patients with PCOS, respectively and 16% had low HDL-C levels.

Insulin resistance, chronic low-grade inflammation, and oxidative stress are wellestablished characteristics of PCOS (^32^), with elevated oxidative markers consistently reported in affected individuals compared to healthy controls (^33^). However, the precise role of mitochondrial dysfunction in the pathophysiology of PCOS remains unclear. This study investigated the differences in MOTS-c levels, one of the MDPs, between adolescents diagnosed with PCOS and the control group. We investigated whether serum MOTS-c levels, one of the MDPs involved in metabolic regulation, differed between adolescents with PCOS and healthy controls. Although serum MOTS-c levels tended to be higher in the PCOS group compared with controls, the difference did not reach conventional levels of statistical significance. To date, only one human study has examined the relationship between MOTS-c and PCOS. That study focused on adult women and examined changes in MOTS-c levels following intralipid and insulin infusion (^14^). At baseline, MOTS-c levels did not significantly differ between women with PCOS and healthy controls. Intralipid infusion increased MOTS-c levels significantly, whereas insulin blunted this response. Moderate exercise over eight weeks had no impact on circulating MOTS-c levels (^14^). Our findings are in line with those reported in adults, suggesting that PCOS may not have a substantial impact on baseline MOTS-c levels during adolescence. On the other hand, a borderline difference in serum MOTS-c levels between PCOS and controls was observed (p = 0.059). Although the study was adequately powered to detect moderate effect sizes based on prior data (^14^), smaller intergroup differences may not have been detected due to limited statistical power. Therefore, this trend should be interpreted cautiously.

Animal studies have demonstrated promising therapeutic effects of MOTS-c, including the prevention of diet-induced obesity, insulin resistance, and fat accumulation (^9^,^11^). These benefits resemble those observed with metformin treatment (^34^). MOTS-c administration has improved insulin sensitivity and glucose metabolism in various experimental models, including gestational diabetes (^35^), autoimmune diabetes (^36^), and ovariectomy-induced obesity (^37^). Despite the robust evidence in animal models, human studies yield inconsistent results, likely due to variations in age, comorbidities, and methodological approaches. For example, Du and cols. (^38^) reported reduced MOTS-c levels in boys with obesity, but not girls, and a negative correlation with obesity markers (BMI, HOMA-IR, hemoglobin A1c (HbA1c), and fasting insulin). In contrast, Cataldo and cols. (^13^) found no difference between adults with obesity and control group but reported a positive correlation between MOTS-c levels and insulin sensitivity individuals with normal weight.

Conflicting associations between MOTS-c levels and metabolic parameters such as BMI, HOMA-IR, and lipid profile suggest a complex, context-dependent role of MOTS-c in humans (^10^,^39^). Some evidence supports the hypothesis that MOTS-c levels rise during the early stages of metabolic imbalance as a compensatory mechanism (^39^), whereas overt metabolic disease (e.g., type 2 diabetes) is associated with decreased MOTS-c levels (^40^). In our study, neither obesity, insulin resistance, nor metabolic syndrome significantly affected serum MOTS-c levels in adolescents with PCOS. Furthermore, no correlation was observed between MOTS-c and metabolic or hormonal parameters, including BMI, glucose, insulin, lipid profile, or androgen levels. Moreover, subgroup analyses according to obesity, insulin resistance, and metabolic syndrome status were exploratory in nature and aimed to further characterize the metabolic heterogeneity of PCOS. Since these comparisons involved secondary analyses rather than independent hypotheses, no formal correction for multiple testing was applied. Therefore, these findings should be interpreted with caution, and future studies with larger cohorts are needed to confirm whether the observed trends represent true biological differences.

Interestingly, MOTS-c levels have been shown to fluctuate based on physiological states. For example, there were elevated levels in pregnant women with obesity and in cases of metabolic syndrome without diabetes (^39^,^41^), whereas levels decreased in type 1 and type 2 diabetes (^10^,^36^) and in individuals with coronary endothelial dysfunction (^42^). Our adolescent cohort may represent an early stage of metabolic disturbance, in which compensatory increases in MOTS-c are still ongoing. Longitudinal studies capturing different disease stages may help to clarify these dynamic patterns. Demonstrating this pattern would provide a clearer insight into the relationship between PCOS and MOTS-c, which is closely linked to obesity, insulin resistance, and metabolic syndrome, which could also help to clarify the pathophysiology of PCOS. Furthermore, MOTS-c levels could serve as a marker for PCOS stages or for monitoring its metabolic effects.

We also explored the potential role of the m.1382A>C (rs111033358) polymorphism in the *MOTS-c* gene, previously associated with type 2 diabetes risk in males from East Asia (^15^,^16^). This SNP, which was found predominantly in haplogroup D4b2, alters the amino acid sequence of *MOTS-c* (K14Q), potentially impairing its biological function. Interestingly, haplogroup D4b2 was associated with longevity (^43^,^44^). However, a larger study reported similar frequencies of the m.1382A>C polymorphism between centenarians and controls, suggesting that this variant is unlikely to contribute substantially to longevity (^16^). In the same study, Japanese male individuals with the m.1382A>C allele variant in the *MOTS-c* gene who are sedentary have an increased incidence of T2DM compared to men with polymorphism who engage in physical activities (^16^). With these results, it has been suggested that the combination of a sedentary lifestyle and the m.1382A> C polymorphism may contribute to an increased risk of type 2 DM (^16^). Moreover, the K14Q MOTS-c treatment of high-fat-fed mice failed to provide the metabolic benefits associated with natural MOTS-c administration, suggesting that the m.1382A>C variant results in inactive endogenous MOTS-c (^11^,^16^). Furthermore, this polymorphism may serve as a biomarker for sex-specific susceptibility to metabolic complications (^45^). Given that the metabolic variability of MOTS-c levels differed by gender in some studies, along with observed gender-specific differences in the m.1382A>C polymorphism, we hypothesized that the *MOTS-c* polymorphism may play a role in PCOS, which is a condition unique to women and characterized by metabolic disturbances. However, in this study, m.1382A>C polymorphism was detected as wild-type (A/A) in all adolescents. The allele frequency of this variant was very low in the Korean population, with C = 0.0520 (https://www.ncbi.nlm.nih.gov/snp/rs111033358). The absence of this variant in our study may be related to the low allele frequency. To the best of our knowledge, this variant was not investigated in patients with PCOS before, so, with this study, we may conclude that the m.1382A>C polymorphism in the *MOTS-c* gene is not a risk factor for developing PCOS. Conversely, the absence of this variant in our study cohort suggests that it may be a population-specific variant. As this polymorphism was originally identified in populations from East Asia, ethnic differences may influence its distribution, and future studies conducted in women with PCOS from this demographic could yield different results.

Another critical finding about MOTS-c studies is that the results can show gender-specific differences. For instance, only male mice were used in the study in which MOTS-c treatment reduced obesity and insulin resistance (^11^). While MOTS-c had no apparent metabolic effect in non-ovariectomized female mice, it prevented metabolic imbalance in ovariectomized mice, suggesting that MOTS-c treatment may prevent postmenopausal obesity and insulin resistance (^37^). In the study previously mentioned, circulating MOTS-c levels were lower in boys and in adolescents with obesity, whereas no difference was observed between girls with and without obesity (^38^). Moreover, the effect of m.1382A>C variant on T2DM is prominent only in male individuals (^16^). These results led to the following claims: the resistance of mitochondrial functions to oxidative stress and antioxidant responses may be related to gender; estrogen activates mitochondrial biogenesis; and ovarian hormones may affect MOTS-c (^16^,^38^). Consistent with previous reports, serum MOTS-c levels were comparable between adolescents with PCOS and healthy peers in our study. Moreover, for the first time, we investigated the relationship between serum MOTS-c concentrations and gonadotropin, estradiol, progesterone, and total testosterone levels in patients with PCOS. No significant associations were observed between MOTS-c and these hormones. These findings suggest that sex-related differences in MOTS-c levels are unlikely to be primarily driven by ovarian or gonadotropic hormones. Large-scale studies involving adolescents, women, and men from diverse ethnic backgrounds are needed to further elucidate these associations.

Several limitations should be considered when interpreting our findings. The main limitations of this study include the absence of metabolic and hormonal profiling in the control group and the evaluation of only a single polymorphism (rs111033358, m.1382A>C) among the *MOTS-c* gene variants. The metabolic and hormonal parameters of the control group were not assessed due to budgetary constraints and ethical considerations regarding extensive blood sampling in adolescents. Although these parameters were presumed to be within the normal range, any unrecognized metabolic or hormonal alterations in controls could have influenced the observed associations. Furthermore, the study was conducted within a specific ethnic population attending a single center. As the prevalence of biochemical or clinical hyperandrogenemia and PCOS differs among ethnic groups, caution is needed when generalizing these findings. Considering that the *MOTS-c* m.1382A>C (rs111033358) polymorphism was first identified in populations from East Asia, further research is needed to evaluate its frequency and potential association with PCOS in diverse ethnic cohorts. Future studies should also include comprehensive metabolic and hormonal profiling of control groups and involve larger, multiethnic populations to enhance the interpretability and generalizability of MOTS-c-related findings.

In conclusion, serum MOTS-c levels were comparable between adolescents with PCOS and healthy controls. Additionally, obesity, insulin resistance, dyslipidemia, and hyperandrogenism did not seem to influence MOTS-c concentrations in adolescents with PCOS. The *MOTS-c* gene m.1382A>C polymorphism was detected as wild-type (A/A) in all participants, suggesting that this variant could not play a role in the etiopathogenesis of PCOS. Taken together, these findings indicate that, in this dataset, we found no evidence of differences in circulating MOTS-c or presence of the m.1382A>C variant related to PCOS. Larger studies in diverse populations are needed to further investigate potential metabolic relevance.

## Data Availability

datasets related to this article will be available upon request to the corresponding author.
